# National Institute for Health Research (NIHR) Health Technology Assessment (HTA) Programme research funding and UK burden of disease

**DOI:** 10.1186/s13063-018-2489-7

**Published:** 2018-02-02

**Authors:** Fay Chinnery, Gemma Bashevoy, Amanda Blatch-Jones, Lisa Douet, Sarah Puddicombe, James Raftery

**Affiliations:** 1 Wessex Institute, University of Southampton, Alpha House, University of Southampton Science Park, Southampton, SO16 7NS UK; 2 National Institute for Health Research (NIHR), Evaluation, Trials and Studies Coordinating Centre (NETSCC), University of Southampton, Southampton, SO16 7NS UK

**Keywords:** Burden of disease, Disability-adjusted life years, National Institute for Health Research, Health Technology Assessment Programme, Funding

## Abstract

**Background:**

HTA Programme funding is governed by the need for evidence and scientific quality, reflecting funding of the National Institute for Health Research (NIHR) by the NHS. The need criterion incorporates covering the spectrum of diseases, but also taking account of research supported by other funders. This study compared the NIHR HTA Programme portfolio of research with the UK burden of disease as measured by Disability-adjusted Life Years (DALYs).

**Methods:**

A retrospective cross-sectional study using a cohort of all funded primary research and evidence syntheses projects received by the HTA Programme from April 2011 to March 2016 (*n* = 363); to determine the proportion of spend by disease compared with burden of disease in the UK calculated using 2015 UK DALY data.

**Results:**

The programme costing just under £44 million broadly reflected UK DALY burden by disease. Spend was lower than disease burden for cancer, cardiovascular and musculoskeletal diseases, which may reflect the importance of other funders, notably medical charities, which concentrate on these diseases.

**Conclusion:**

The HTA Programme spend, adjusted for other relevant funders, broadly matches disease burden in the UK; no diseases are being neglected.

**Electronic supplementary material:**

The online version of this article (10.1186/s13063-018-2489-7) contains supplementary material, which is available to authorized users.

## Background

Comparing funding to the burden of disease, as measured by Disability-adjusted Life Years (DALYs), has been used to assess the appropriate spread of spending. DALYs take into account both the potential years of life lost due to premature death and equivalent years of healthy life lost by virtue of being in states of poor health or disability [1]. Previous studies comparing United States (US) National Institutes of Health (NIH) funding to burden of disease have shown DALYs to be the single best correlate with spend by disease [2]. Other studies have focussed on the association between randomised trial or systematic review evidence and global burden of disease [3–5].

**Fig. 1:**
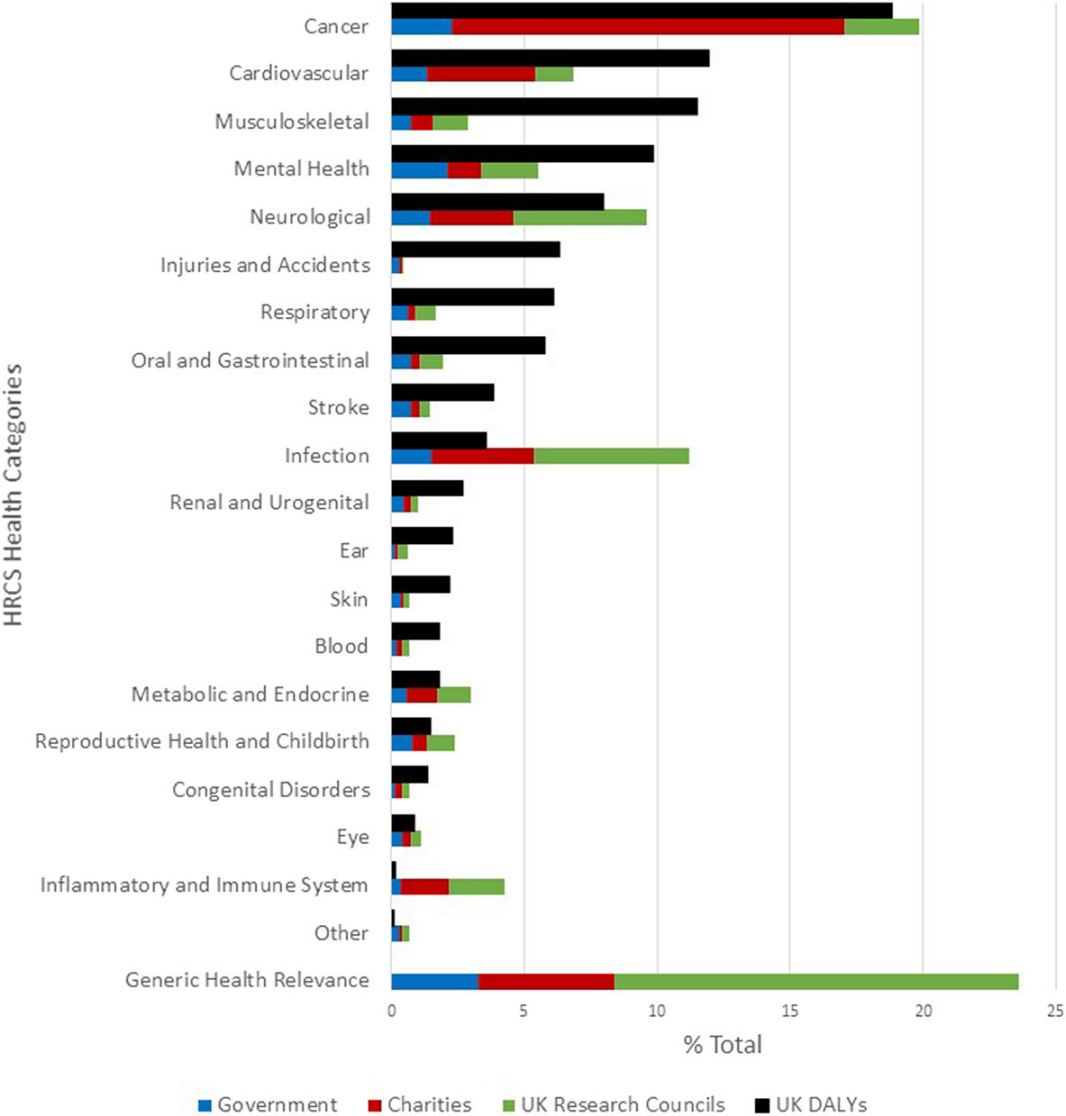
UK public-funder spending compared with UK burden of disease (Disability-adjusted Life Years; DALYs) mapped to Health Research Classification System (HRCS) health categories

The UK Clinical Research Collaboration (UKCRC) undertook an analysis of public funding of health relevant research in the UK in 2014 [6]. The analysis included funding by 64 organisations such as the government, charities and UK Research Councils (RCUK), corresponding to over £2 billion of investment. Figure 1 summarises the UKCRC results, by descending DALYs. Of the four disease categories with the largest DALY burden, only public spend on the Cancer category is closely correlated with the burden of disease in the UK, and the majority of this support is provided by charities.

Funding by the Department of Health (UK government) covered 29 departments, but most of the funding was for infrastructure, rather than research. The National Institute for Health Research (NIHR) Health Technology Assessment (HTA) Programme is included among the 29 Department of Health departments. The HTA Programme is the largest and longest-running NIHR research programme, it mainly funds research trials and systematic reviews of the clinical and cost-effectiveness, of health technologies.

Criteria for HTA Programme funding are (1) the need for evidence and (2) scientific quality, reflecting funding of the NIHR by the NHS [7]. The need criterion has two implications: coverage of the spectrum of diseases, but also taking account of research supported by other funders. The latter include the UK’s disease-specific medical charities, the biggest of which cover cancer, cardiovascular and musculoskeletal diseases. Consequently, one would expect HTA Programme spend to be well spread across diseases, but relatively lower in those diseases which have substantial charity funding. This paper compares HTA Programme spend with the UK burden of disease as an indicator of ‘need’.

## Methods

### Data source and classification of disease

Age-standardised 2015 DALY data for the UK (all ages and both sexes) from the Institute for Health Metrics and Evaluation Global Health Data Exchange were used [8]. These data for 316 specific diseases were assigned to the 21 Health Research Classification System (HRCS) health categories (which are derived from the World Health Organisation International Classifications of Diseases) independently by two of three coders (GB, LD and AB-J; data available in Additional file 1). Any disagreements were resolved in discussion with the third coder. The nature of the HRCS prevents dual coding. If a disease falls into two or more HRCS categories, the disease is apportioned equally between the two codes. For example, cellulitis is coded as 50% Infection and 50% Skin in HRCS. This coding gave a DALY estimate for each health category, allowing the percentage of the total burden of disease in the UK for each category to be calculated.

### Sample selection

All funded primary research and evidence syntheses projects received by the HTA Programme between 1 April 2011 to 31 March 2016 were included (*n* = 363; data available in Additional file 1).

All funded applications received by the HTA Programme are routinely coded using the HRCS. If the disease described in an application falls into two different HRCS categories, the project is apportioned equally between the assigned codes. For example, a project concerning diabetic retinopathy would be coded as 50% Eye and 50% Metabolic and Endocrine.

### Data analysis

HTA Programme spend data were derived from the NIHR Evaluation, Trials and Studies Management Information System (NETS MIS) and mapped to DALY data using HRCS health categories. All analyses were in Excel 2013.

Costs included research costs only.

## Results

**Fig. 2:**
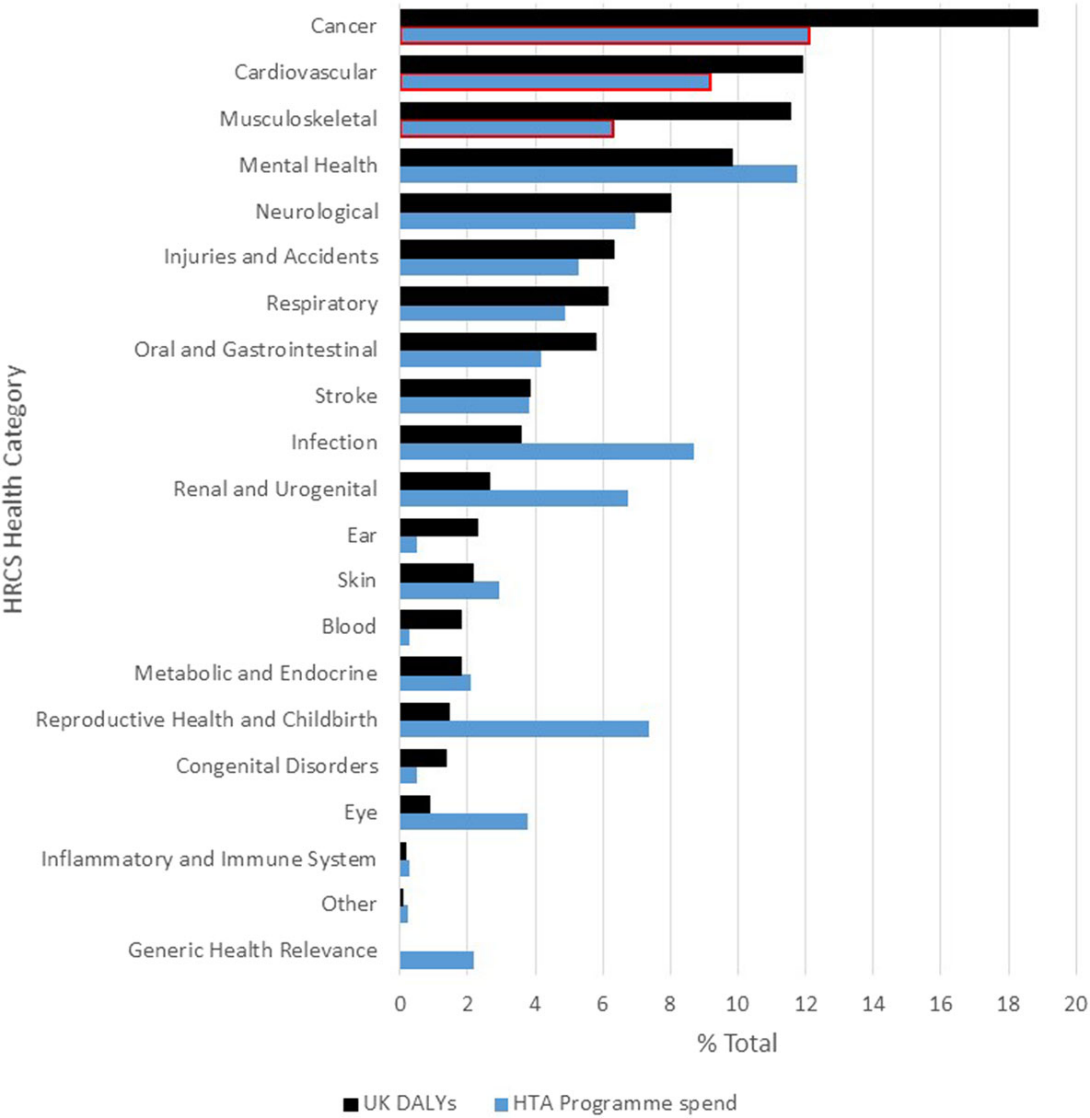
National Institute for Health Research (NIHR) Health Technology Assessment (HTA) Programme spend compared with UK burden of disease (Disability-adjusted Life Years; DALYs) mapped to Health Research Classification System (HRCS) health categories

Three hundred and sixty-three projects were identified for analyses; 277 were randomised controlled trials (RCTs). Total spend was just under £400 million and RCT spend was £354 million (Table 1). HTA Programme total spend (by HRCS category) was compared with 2015 UK DALYs (Fig. 2).

**Table 1:** National Institute for Health Research (NIHR) Health Technology Assessment (HTA) Programme total spend and randomised controlled trial (RCT) spend compared with the burden of disease (Disability-adjusted Life Years; DALYs) mapped to Health Research Classification System (HRCS) health categories

HRCS health category	Total apportioned spend (£)	RCT apportioned spend (%)	DALY burden (%)
Cancer	48,141,582	43,289,092	18.84
Cardiovascular	36,492,300	32,422,740	11.92
Musculoskeletal	24,978,542	21,728,235	11.53
Mental health	46,745,011	43,758,644	9.83
Neurological	27,610,923	26,963,249	7.99
Injuries and Accidents	21,045,089	19,834,241	6.33
Respiratory	19,322,359	14,646,317	6.12
Oral and Gastrointestinal	16,654,803	14,152,800	5.79
Stroke	15,093,925	14,841,635	3.84
Infection	34,490,269	30,806,401	3.58
Renal and Urogenital	26,762,773	23,925,063	2.66
Ear	1,947,835	1,243,473	2.31
Skin	11,626,036	11,527,412	2.16
Blood	1,178,448	1,097,847	1.80
Metabolic and Endocrine	8,287,045	6,602,431	1.79
Reproductive health and Childbirth	29,248,437	26,391,283	1.45
Congenital disorders	1,928,151	1,463,336	1.35
Eye	14,971,063	10,276,840	0.89
Inflammatory and Immune	1,069,161	1,069,161	0.16
Other	994,430	994,430	0.08
Generic health relevance	8,623,682	7,281,761	0.00
Total	397,211,864	354,316,391	100

Total spend across the 21 health categories was fairly well correlated with their DALY burden, (simple linear regression; *r*^2^ = 0.61; 95% confidence interval (CI) 0.36–0.82).

Cancer (19%), Cardiovascular (12%) and Musculoskeletal (12%) categories represent the largest disease burden in the UK, but the proportion of HTA Programme spend was considerably lower than the associated DALY percentage (Fig. 2). Disease areas well supported by the medical charities are marked by a red border in Fig. 2 indicating that any shortfalls in these disease categories were to some extent compensated for by the medical charities

Spend by the HTA Programme was higher in Mental health, Infection, Reproductive health and Childbirth, Renal and Urogenital and Eye categories than their associated DALY percentage (Table 1).

## Discussion

HTA Programme spend broadly supports DALY burden by disease in the UK; no diseases are being overlooked, either in terms of total spend or from the perspective of RCT spend (Additional file 2). Discrepancies between DALYs and spend by the HTA Programme are compatible, with the latter funding less research in diseases supported by the medical charities.

### Strengths and limitations

The main strength of the study was the inclusion of 363 research projects over a 5-year period. This reduced the variation that could otherwise be caused by funding a few expensive projects in a short period of time, or the effects of NIHR research priority calls which could cause spikes in certain disease areas.

A limitation of the study was the use of DALYs, which are crude measures of overall disease burden, and do not capture the wider effects of interventions such as Quality of Life, impact on carers and family, or non-health effects such as economic and social consequences (such as loss of work) [9, 10].

### Implications

As expected, there was a lower proportion of HTA Programme spend for cancer, cardiovascular and musculoskeletal diseases. These three disease categories have funding by three large medial charities: Cancer Research UK, British Heart Foundation (BHF) and Arthritis Research UK. The largest medical charity, the Wellcome Trust, supports research across all diseases [6].

There were a number of diseases where HTA Programme spend was greater than the burden of disease, including Infection, Reproductive health and Childbirth, Renal and Urogenital and Mental health categories. These research topics have been neglected historically. For example, national and global underinvestment in mental health and infectious diseases has been noted [11, 12].

The relationship between DALY data, allocation of research funding and improvements in health is likely to be complex because health problems are not all equally set for research advances [13]. Diseases vary by the existence of interventions that might be usefully evaluated.

Increased research funding for a disease may not automatically reduce the burden of disease. An analysis of NIH funding and its correlation with US health dynamics over 50 years found that increased funding for heart disease and stroke was associated with reduced burden of disease; but there were no clear associations for cancer and diabetes [14]. In addition, spend may not be the most appropriate measure to compare with burden of disease because projects vary in cost, with trials costing much more than systematic reviews.

## Strengths and limitations

- A large cohort of 5 years’ of research funded by a major funder of health research in the UK
- DALYs only partly capture the effects of interventions on Quality of Life, on carers and family, and economic and social consequences (e.g. loss of work)

## Conclusions

DALY analysis is a useful screen to see if any disease areas are being neglected, and so is of value when looking at the spread of funding by individual funders. Our results suggest that there are no major concerns for the spread of HTA Programme spending.

Other funders may find it useful to perform a similar DALY analysis. However, funding organisations should not align their spend directly against disease burden using DALYs, given that there may be a lack of worthwhile interventions to test in some diseases, and that research may already be well supported by other organisations.

## Additional files

Additional file 1: 2015 DALYs coded to HRCS and HTA programme spend raw data. (XLSX 101 kb)

Additional file 2: HTA programme RCT spend. (JPEG 75 kb)
