# Supplementary figures and images for: National Institute for Health Research (NIHR) Health Technology Assessment (HTA) Programme research funding and UK burden of disease
Source: Trials. 2018 Feb 2;19:87. doi: 10.1186/s13063-018-2489-7 (PMC5797405; doi:10.1186/s13063-018-2489-7)

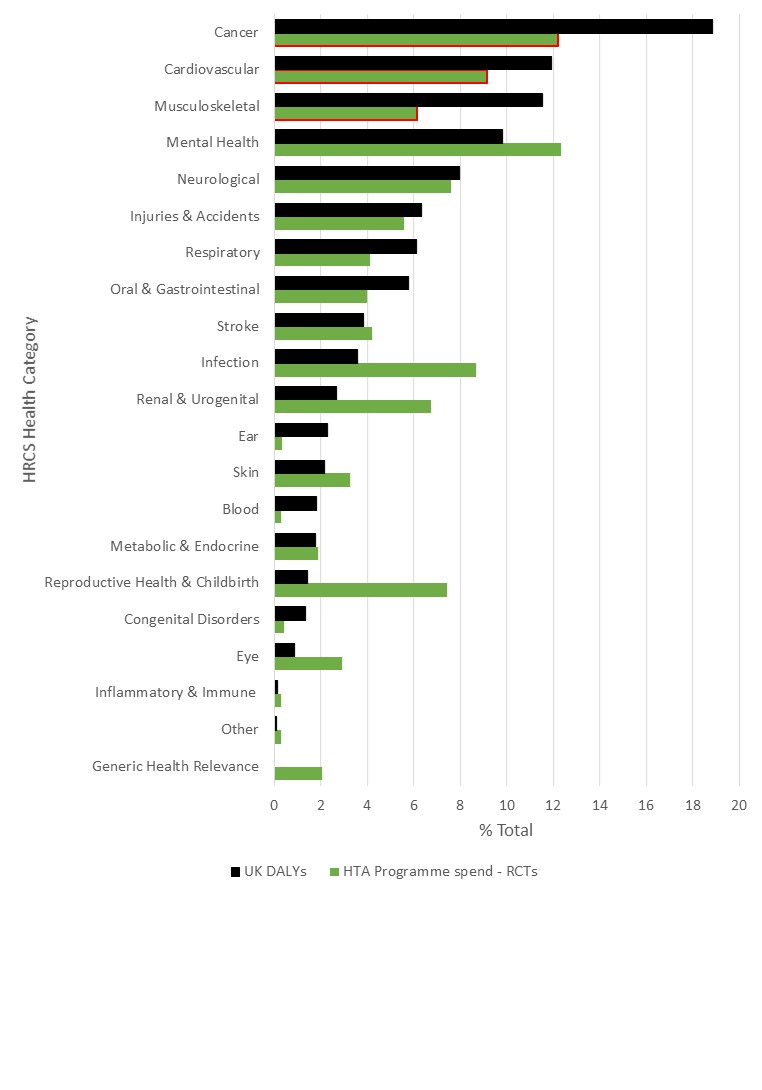

Supplement: Supplementary file 2 — HTA programme RCT spend. (JPEG 75 kb) [file 13063_2018_2489_MOESM2_ESM.jpg]
